# Observations upon the Sensations of Taste and Smell

**Published:** 1812-12

**Authors:** 


*To the Editors of the Medical and Physical Journal.*

GENTLEMEN,

AS many of the following observations are not, I believe, to be met with in the works of any physiological writer, should they be deemed sufficiently interesting to merit a place in your valuable miscellany, the insertion of them will gratify, Gentlemen,

Your most obedient Servant,

W. P.

FLAVOR is defined, by most authors,* to be that quality or principle in a substance which is capable of exciting the sense of taste. How far this is consonant to the original meaning of the term, I do not pretend to determine; but in common language, nothing is more frequent than to hear it used in a sense apparently synonymous with taste: thus it is said, indiscriminately, such a thing has a bitter *taste*, or *flavor*, &c. That they are not, however, synonymous, will, I think, appear to every one upon a moment's reflection; indeed, the truth is, that they represent qualities† of substances, or rather sensations produced by them totally distinct from each other.

To endeavor to point out in what this difference consists, is the object of the following remarks; and, as flavor seems to partake both of the nature of taste and smell, I shall premise some observations on each of these senses.

*Taste* is that sensation which is produced by substances when brought into contact with the tongue under certain circumstances, *the nostrils being at the same time closed.*

The circumstances alluded to, and which appear to be absolutely necessary to the production of this sense, are, first, that the tongue be moist; secondly, that the substances be more or less soluble in water; and thirdly, motion.

---

* See Richerand, &c.

† Perhaps in all languages the same term represents both the *sense* of taste, and the *qualities* of substances capable of exciting that taste.

Of these, the first is universally admitted: it is also well known, that a substance capable of exciting taste must be more or less soluble in water, or the saliva. That many of the metals excite taste, is no argument against this position, since the property is confined to those easily oxidized: in a word, taste appears to be universally produced by, or during the moment of, chemical action, especially when this is effected, as perhaps it is in every instance, by galvanic agency.

Motion appears to be necessary to the production of this sense; for, if we apply any substance to the tongue in such a manner that it may be at rest, and its action circumscribed, the sense of taste will be very imperfectly, if at all, produced. The moment, however, it be moved about on the tongue, and more especially if the tongue be applied with it to any part of the mouth, we perceive the taste very distinctly, and this we do involuntarily when we wish to ascertain the taste of a substance.

In general, substances do not excite taste, that are not capable of exciting common sensation when applied to a part of the body denuded of the cuticle; and hence it is that salts form so large a proportion of sapid substances. The class of bitters, however, are, in a certain degree, an exception to this.

The seat of taste is chiefly confined to the upper surface of the extremity of the tongue. The under surface, however, is not destitute of the power of tasting, as any one may convince himself, by moistening the lower lip with any sapid solution, and then applying the lower surface of the tongue in contact with it.

*Smell* is that sensation which is produced by substances when they are brought into contact, under particular circumstances, with the internal membrane of the nose.

As in taste, so in smell, three circumstances appear to be necessary: first, that the nose be moist; secondly, that the substance be soluble in *air; and thirdly, motion.

The two former of these being generally admitted, I shall confine my observations to the latter.

The necessity of motion is evident, from the fact that we can, by merely holding the breath without closing the nose,

* The quality of the air, so that it does not decompose the odorous substance, seems not in the least to influence the process of smelling: thus I tried oxygen, hydrogen, nitrogen, and carbonic acid, gases, as the vehicles for odors, without the least sensible difference, except in the case of oxygen, which seemed rather to affect the sensation produced by some odors, probably from combining with them.

suspend suspend the sense of smell at pleasure, though surrounded by air containing odorous substances in solutions. This may be rendered still more striking by holding a phial to the nose, containing any odorous substance, at the same time suspending inspiration; in this case none of the odor will be perceived, but, after having removed the phial, and walked into another part of the room where the odor does not exist, and then drawing in the breath, it will be plainly perceived; an evident proof that it exists all this while in the nose, though it does not excite sensation.

From the peculiar structure of the nose, it is almost impossible to say what part of it is the precise seat of smell. It seems however probable, from the above circumstances, that those parts only against which the air comes in contact during its passage through the nose can possess this faculty; and hence that the sinuses, through which there is no circulation of air, can have no share in the production of this sense, as some have supposed.

In treating of *flavor*, perhaps I cannot introduce the subject better than by relating the circumstance that first drew my attention to it; in fact, this is an act of justice due to the eminent character with whom it originated.*

It is now many years ago since I heard it asserted "that a nutmeg has no taste," and witnessed a position so apparently paradoxical proved to be correct. The experiment was directed to be made *with the nostrils closed*, and in this case it was found to be impossible to distinguish, by what is properly called the *taste*, whether a portion of nutmeg, or any other substance, was introduced into the mouth. The experiment I often thought of afterwards, and repeated, not only with the nutmeg, but with many other substances, and at length was induced to adopt the opinion which I have here to advance, viz. that the sensation produced by the nutmeg or any other substance, when introduced into the mouth, and which ceases the moment the nostrils are closed, is really very different from taste, and ought to be distinguished by

* Of course an anonymous writer cannot mention names, I shall therefore only observe, that the gentleman here alluded to, at present discharges the arduous duties of a public situation with infinite honor to himself, and at the same time is not more distinguished for the excellence of his head than of his heart. The same reasons also prevent me mentioning the name of another eminent character, for whom I shall ever entertain the most profound respect, who long time ago gave his general assent to what I here advance.

another name; that that name should be *flavor*, the one which seems most naturally and properly to designate it.

After this the definition I have given of taste, and also the following one of flavor, will be understood.

*Flavor* is that sensation which is produced when substances under certain circumstances are introduced into the mouth, *the nostrils being at the same time open.*

As this sense seems to be of an intermediate nature between taste and smell, it requires all the circumstances common and peculiar which have been stated to be necessary to the production of these two senses.

Almost all substances produce more or less of *flavor*, but those in which this power seems more particularly to reside, are such as contain a principle *soluble* more or less *both in water and air*, especially the latter; and hence all substances containing a volatile oil, as the various spices, aromatics, &c. have this power in an eminent degree, though they excite comparatively very little taste. Ammonia, however, (and perhaps some other substances,) may be considered as an exception to the above, since it excites little more flavor than a common salt; this may probably be owing to its very great affinity for water.

I once thought that the mere perviousness of the nose, without any notice of the air through it, was sufficient for the production of this sense; but I am now convinced that this notion was not accurate, and that, during the act of *flavoring*, if I may be allowed the expression, there is a slow *expiration* of the air through the nostrils, and at the same time generally a slow *inspiration* through the mouth.

The seat of flavor is much more extensive than that of taste, or perhaps of smell, at least in the human species, as it seems to occupy the whole of the mouth, in a greater or less degree, also the fauces, back part of the nose, &c.

In short, from these and many other circumstances that might be adduced, I am inclined to believe, that flavor is an intermediate sensation partaking both of the nature of taste and smell; that the properties in which it partakes of the nature of each, varies in different instances; but that it always partakes more of the nature of smell than of taste.

Admitting what has been advanced, we can easily account for the circumstance of a person's being able to taste, as it has been called, who has been born without a tongue, of which there are many instances on record. We can also explain the vulgar error, viz. that a person who loses the sense of smell, necessarily loses that of taste, &c. The sense of taste can, however, exist independently of smell, as I am acquainted

 with with a person who has lost his smell, and consequently his *flavor*, but who enjoys the power of taste very perfectly.

Much more might be said on this curious subject, but, as I have already intruded too much on the patience of my readers, I shall here stop for the present, intending at some future time, should this meet with a favorable reception, to resume the subject.

